# Adenosine inhibits TNFα-induced MMP-3 production in MH7A rheumatoid arthritis synoviocytes via A_2A_ receptor signaling

**DOI:** 10.1038/s41598-022-10012-6

**Published:** 2022-04-11

**Authors:** Hiroe Konishi, Shun-En Kanou, Rika Yukimatsu, Mizuki Inui, Motoya Sato, Naruto Yamamoto, Masayoshi Nakano, Masahiro Koshiba

**Affiliations:** 1 Department of Clinical Laboratory Medicine, Hyogo Medical University School of Medicine, 1-1 Mukogawa-cho, Nishinomiya, Hyogo 663-8501 Japan; 2 Department of Clinical Laboratory, Hyogo Medical University Hospital, Nishinomiya, Hyogo 663-8501 Japan

**Keywords:** Immunology, Tumour-necrosis factors

## Abstract

Adenosine causes the anti-inflammatory effect of MTX; however, the contributions of synoviocyte adenosine receptors (AdoRs) are unknown, and matrix metalloproteinase 3 (MMP-3) is released by fibroblast-like synoviocytes in response to inflammatory signaling. To understand the mechanism of the clinical observation that the matrix proteinase-3 concentration of patients with rheumatoid arthritis treated successfully with methotrexate does not usually normalize, we investigated the effects of A_2A_ AdoR activation and inhibition on tumor necrosis factor-alpha (TNFα)-induced MMP-3 release by MH7A human rheumatoid synovial cells. MH7A cells constitutively expressed membrane-associated A_2A_ AdoRs, and HENECA enhanced intracellular cAMP. Stimulation with TNFα markedly enhanced release of MMP-3 from MH7A cells, whereas HENECA partially and dose-dependently inhibited TNFα-evoked MMP-3 release. Similarly, dbcAMP partially inhibited TNFα-induced MMP-3 release. Pretreatment with ZM241385 reversed the inhibitory effects of HENECA. Further, TNFα induced p38 MAPK and ATF-2 phosphorylation, whereas HENECA suppressed p38 MAPK and ATF-2 phosphorylation. We concluded that adenosine signaling via A_2A_ AdoRs, adenylyl cyclase, and cAMP reduces TNFα-induced MMP-3 production by interfering with p38 MAPK/ATF-2 activity. Activation of A_2A_ AdoR signaling alone using HENECA did not reduce TNFα-induced MMP-3 production to the basal levels, which may explain why MTX usually decreases but does not eliminate serum MMP-3.

## Introduction

Rheumatoid arthritis (RA) is a chronic inflammatory disorder characterized by joint pain, stiffness, and immobility, typically starting in smaller peripheral joints and eventually afflicting the larger joints^1^. While the underlying etiology is still unclear, these symptoms are associated with inflammatory proliferation of synovial cells induced by cytokines released from infiltrating lymphocytes and macrophages, eventually resulting in pannus formation and joint destruction^2,3^.

Matrix metalloproteinases (MMPs) are a family of enzymes that catalyze the degradation of extracellular matrix. Most MMPs are secreted as inactive preproteins that are activated when cleaved by extracellular proteinase^4^. The human enzyme MMP-3, also known as stromelysin-1, is expressed and secreted by fibroblast-like synoviocytes (FLSs) and chondrocytes within joints upon stimulation by inflammatory cytokines such as TNFα^5^. Once released and activated, MMP-3 degrades a wide variety of extracellular matrix proteins, including collagen types II, III, IV, IX, and X, proteoglycan, fibronectin, laminin, and elastin^6^. Moreover, MMP-3 can activate several other MMPs, including MMP-1, MMP-7, and MMP-9, thereby amplifying matrix proteolysis^7^. It is thought that these proteases contribute to joint destruction in RA by degrading cartilage extracellular matrix^8,9^. Indeed, joint MMP-3 concentration is markedly elevated in RA compared to other MMPs^8,10^. Further, increased production by FLS cells is frequently associated with a parallel increase in serum MMP-3 concentration. Elevated serum MMP-3 is observed in 80%–90% of RA patients, and reflects the degree of synoviocyte proliferation. Thus, serum MMP-3 may be a prognostic indicator of disease progression, especially in the early phase of RA^11^. When serum MMP-3 concentrations are high, joint destruction is expected to progress rapidly. Conversely, MMP-3 levels decrease when the condition stabilizes in response to antirheumatic drugs, including biologics^12^.

Methotrexate (MTX) is recommended as a first-line drug for the initial treatment of RA^13,14^. The antirheumatic effects of MTX are thought to be mediated by both inhibition of dihydrofolate reductase^15,16^ and by increasing the release of adenosine^17–20^. Dihydrofolate reductase is an enzyme that produces tetrahydrofolate required for nucleic acid synthesis, resulting in the suppression of immunocyte proliferation and enhanced apoptosis^17,18^. Adenosine is a purine metabolite produced by most cells and required for adenosine triphosphate (ATP) synthesis. Further, adenosine acts as an intercellular signaling factor by stimulating four G protein-coupled adenosine receptors (A_1_, A_2A_, A_2B_, and A_3_), all of which are expressed on synoviocytes^21,22^. Among these, A_2A_ AdoR signaling via the G protein Gs has been shown to stimulate cAMP formation via adenylyl cyclase activation, which antagonizes T cell receptor signaling^23,24^. In addition, intracellular cAMP may modulate MMP-3 expression^25^. RA patients treated with MTX exhibited reduced blood MMP-3 levels^26^, suggesting that adenosine negatively regulates MMP-3 production by FLSs. However, the specific functions of AdoRs expressed on synovial cells have not been clarified. We therefore investigated how adenosine A_2A_ AdoR signaling affects TNFα-induced MMP-3 production in an RA FLS cell line (MH7A).

## Results

### Detection of functional A_2A_ AdoR expression in MH7A cells

**Figure 1:**
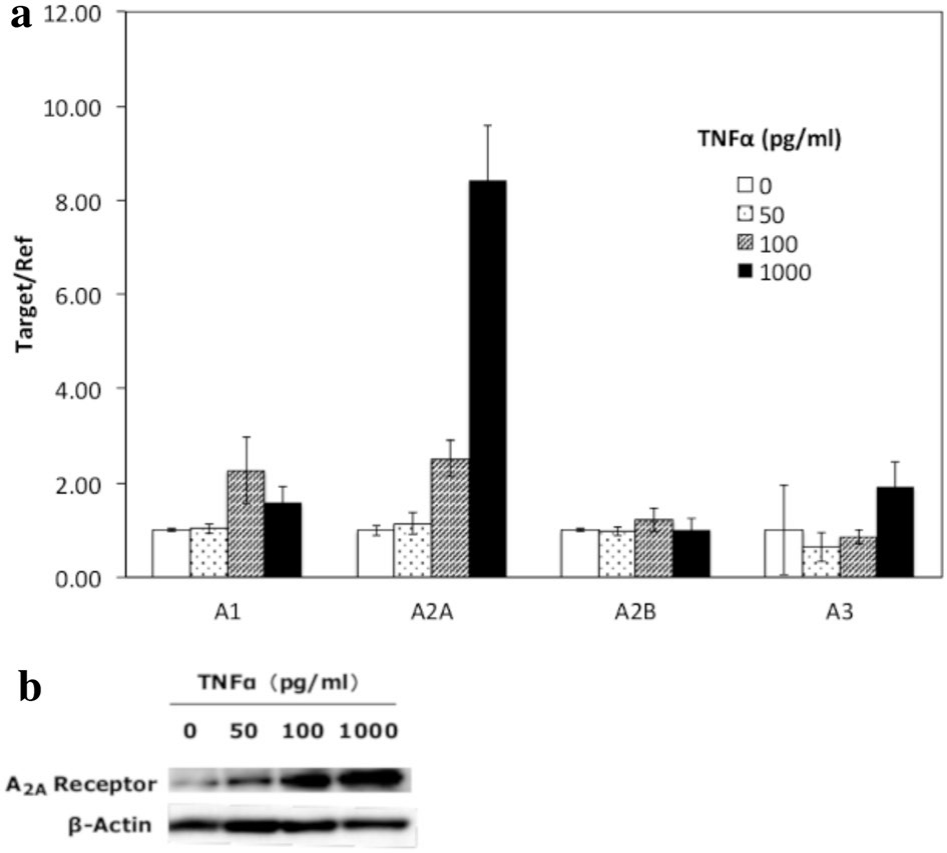
Expression of all four adenosine receptors by MH7A cells. Expression of adenosine A_2A_ receptors in MH7A cells and enhancement by TNFα. MH7A synoviocytes were incubated for 24 h with the indicated concentrations of TNFα. Adenosine receptor (AdoR) mRNA levels and A_2A_ AdoR protein in the membrane fraction were then determined by real-time PCR (**a**) and Western blotting (**b**), respectively. Both A_2A_ AdoR mRNA and membrane protein expression were enhanced by TNFα compared to untreated controls. ß-actin was used as an internal control. Original blots/gels are presented in Supplementary Figs. 1, 2. Experiments were repeated three times and representative data are shown.

**Figure 2:**
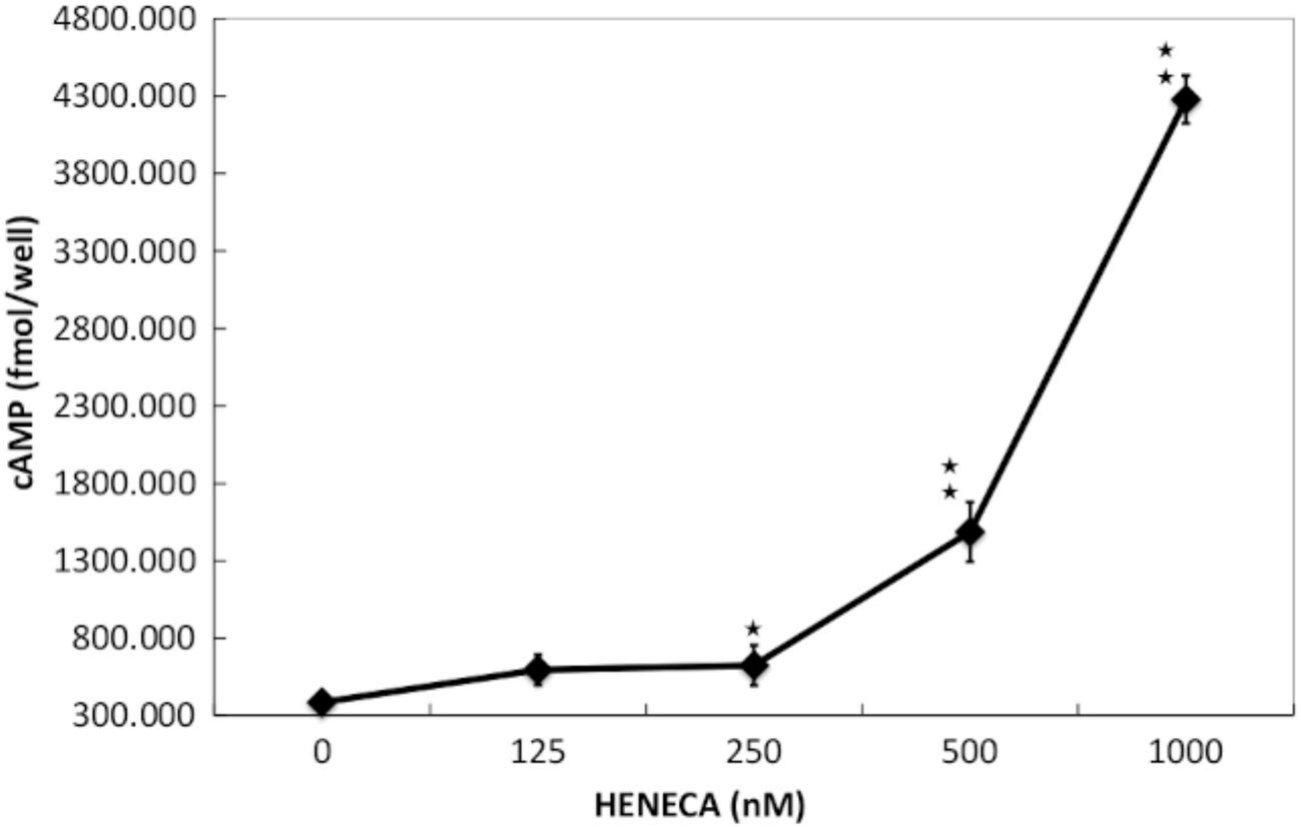
Detection of functional A_2A_ AdoR expression in MH7A cells. Adenosine A_2A_ receptors in MH7A cells are functionally coupled to adenylyl cyclase and intracellular cAMP production. Cells were stimulated for 30 min with the indicated concentration of A_2A_ AdoR agonist HENECA. Experiments were repeated three times, and data are presented as the mean ± SD. ^★^*p* < 0.01 versus 0 μM HENECA. Intracellular cAMP levels were measured using an enzyme immunoassay.

Combined RT-PCR and western blotting revealed the expression of all four adenosine receptor mRNAs and proteins by MH7A cells. Further, expression of both A_2A_ AdoR mRNA in cytoplasm and A_2A_ AdoR protein in the membrane fraction were increased in a concentration-dependent manner by TNFα stimulation (Fig. 1). The A_2A_ AdoR agonist HENECA also induced a concentration-dependent increase in intracellular cAMP (Fig. 2), indicating that these membrane receptors were functionally coupled to adenylyl cyclase (AC) via Gs.

### Stimulation of A_2A_ AdoRs reduced basal and TNFα-induced MMP-3 expression and release from MH7A cells

**Figure 3:**
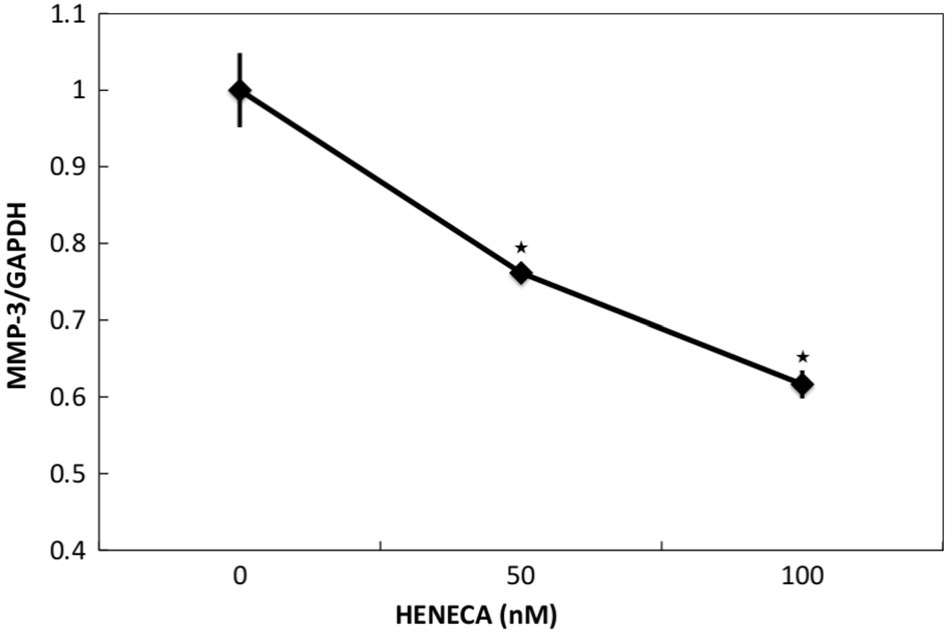
Stimulation of A_2A_ AdoRs reduced the basal and TNFα-induced MMP-3 expression by MH7A cells. Activation of A_2A_ receptors suppressed constitutive expression of MMP-3 mRNA in MH7A cells. Cells were incubated for 24 h with the indicated concentration of HENECA, and MMP-3 mRNA levels estimated by RT-PCR. Experiments were repeated three times, and data are presented as the mean ± SD. ^★^*p* < 0.05 and ^★★^*p* < 0.01 vs. 0 μM HENECA.

**Figure 4:**
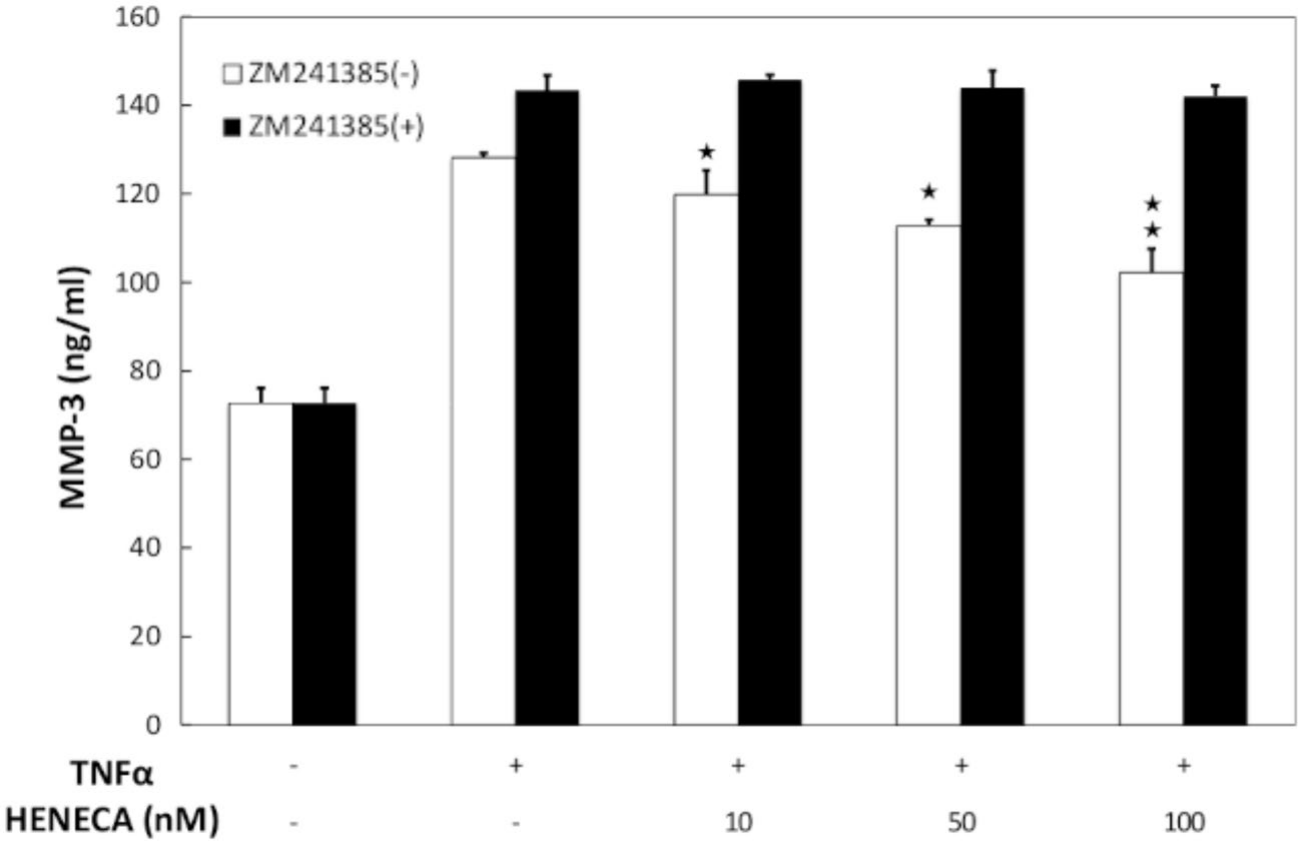
Stimulation of A_2A_ AdoRs reduced the basal and TNFα-induced MMP-3 release by MH7A cells. Activation of A_2A_ receptors suppressed TNFα-induced MMP-3 production by MH7A cells. MH7A cells were incubated for 24 h with TNFα (25 pg/ml) with or without the indicated concentrations of HENECA (white bars). In some experiments, the cells were also pretreated for 30 min with 1 μM of the selective A_2A_ AdoR antagonist ZM241385 (black bars). MMP-3 was then measured in the culture medium. The suppressive effect of HENECA on TNFα-induced MMP-3 production was blocked by ZM241385 pretreatment. Experiments were repeated three times, and data are presented as mean ± SD. ^★^*p* < 0.05 and ^★★^*p* < 0.01 versus TNFα alone.

**Figure 5:**
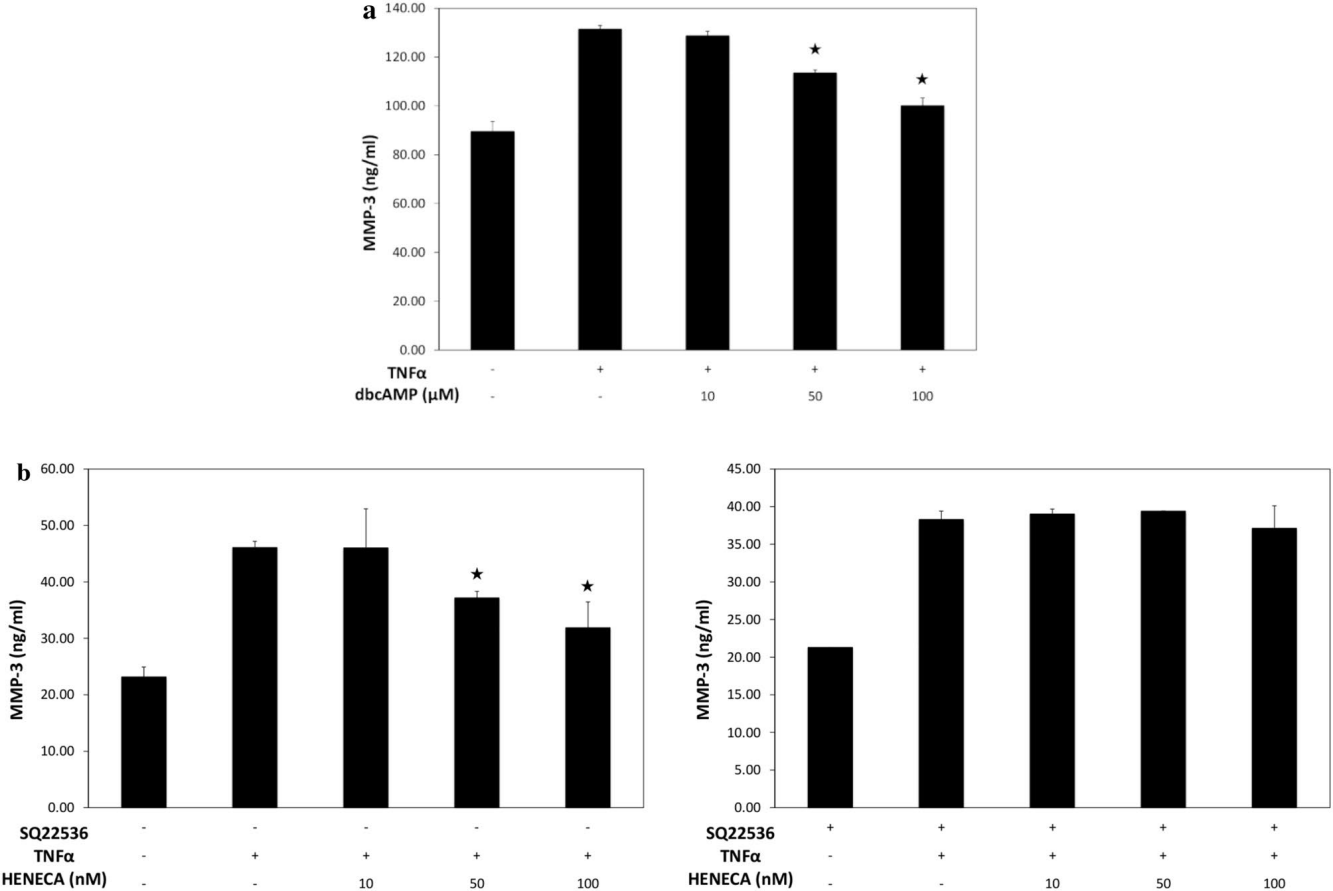
Effect of cAMP analog dbcAMP and the adenylate cyclase inhibitor SQ22536 on TNFα-induced enhancement of MMP-3 production. Activation of cAMP signaling suppressed TNFα-induced MMP-3 production (**a**). MH7A cells were incubated for 24 h in TNFα (25 pg/ml) with or without the indicated concentration of dbcAMP, and MMP-3 concentration measured in the culture medium. In some experiments, the cells were also pretreated for 30 min with 0.1 mM of the adenylate cyclase inhibitor SQ22536 (**b**). MMP-3 was then measured in the culture medium. The suppressive effect of HENECA on TNFα-induced MMP-3 production was blocked by SQ22536 pretreatment. Experiments were repeated three times and data are presented as mean ± SD. ^★^*p* < 0.01 vs. TNFα alone.

In accord with previous studies^27,28^, MH7A cells constitutively expressed MMP-3 mRNA, and expression was reduced dose-dependently by HENECA (Fig. 3). In addition, MMP-3 protein release into the extracellular medium was accelerated by TNFα stimulation (Fig. 4, white bars). This response was partially inhibited in a concentration-dependent manner by co-treatment with HENECA (*p* < 0.05 at 10 or 50 nM HENECA and *p* < 0.01 at,100 nM HENECA vs. TNFα alone), consistent with HENECA-induced suppression of MMP-3 mRNA expression (Fig. 3). Pretreatment of MH7A cells with the A_2A_ AdoR antagonist ZM241385 blocked the inhibitory effects of HENECA on TNFα-induced MMP-3 release (Fig. 4, black bars). Consistent with a contribution of A_2A_ AdoR/Gs/AC signaling to these responses, the cAMP analog dbcAMP also blocked TNFα-induced augmentation of MMP-3 production (*p *< 0.01 at 50 or 100 μM dbcAMP vs. TNFα alone) (Fig. 5a). Additionally, the pretreatment of MH7A cells with the adenylate cyclase inhibitor SQ22536 blocked the inhibitory effects of HENECA on the TNFα-induced MMP-3 release (Fig. 5b). Collectively, these results indicate that adenosine signaling via the A_2A_ AdoR/Gs/AC/cAMP pathway can block TNFα-mediated MMP-3 production in MH7A synoviocytes.

### Stimulation of A_2A_ AdoRs reverses TNFα-mediated activation of p38 MAPK signaling in MH7A cells

**Figure 6:**
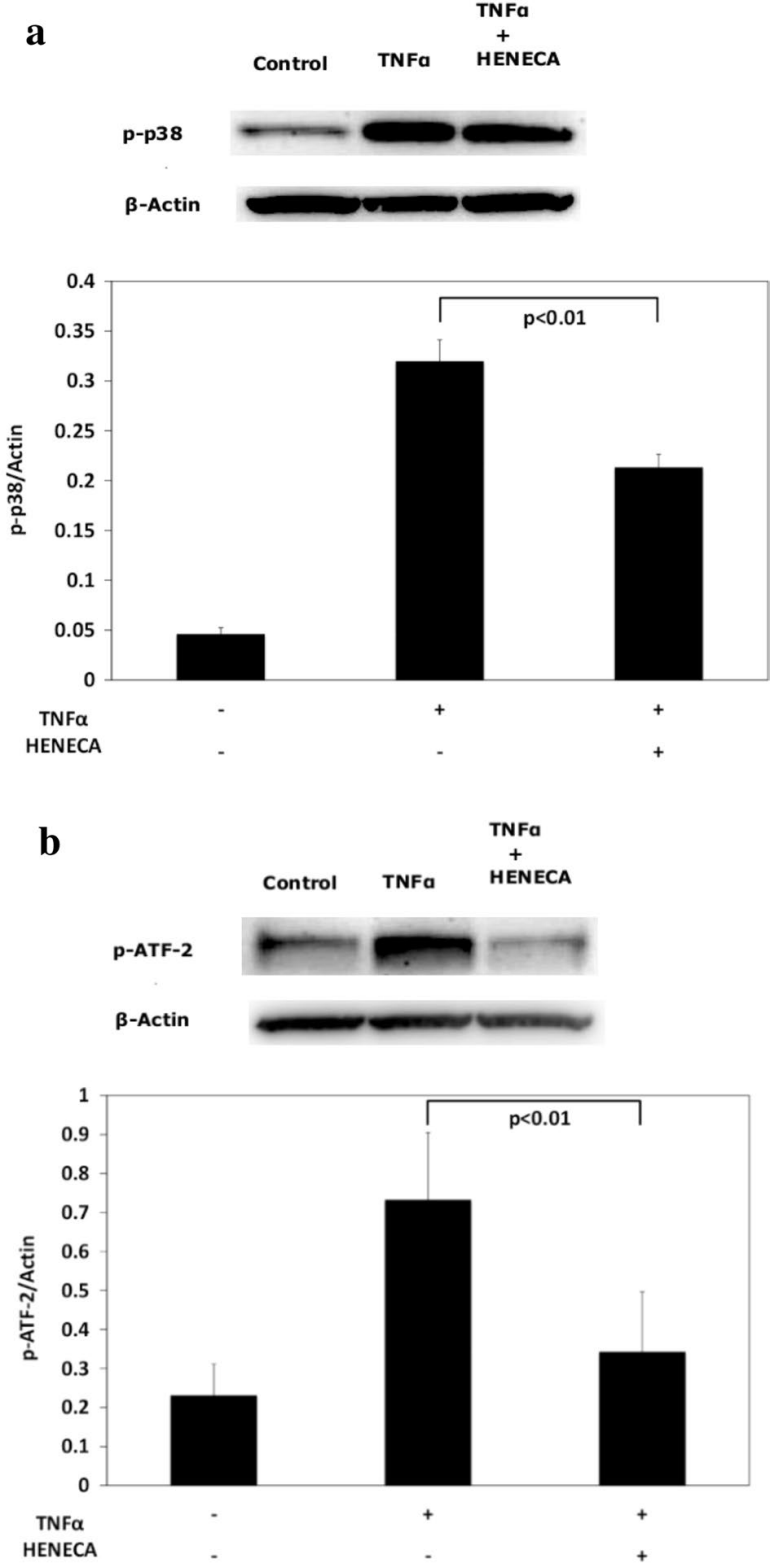
Stimulation of A_2A_ AdoRs reverses the TNFα-mediated activation of p38 MAPK signaling in MH7A cells. Activation of A_2A_ receptor signaling suppressed TNFα-induced activation of p38 MAPK and ATF-2. Cells were incubated for 10 min (**a**) or 1 h (**b**) in TNFα (1 ng/ml) with or without HENECA (1 μM). Expression levels of p-p38 and p-ATF-2 were estimated by western blotting and densitometry. HENECA suppressed TNFα-induced phosphorylation (activation) of p38 MAPK (**a**) and ATF-2 (**b**). ß-actin served as the gel loading control. Original blots/gels are presented in Supplementary Figs. 3, 4, 5, 6. Experiments were repeated three times and data are presented as the mean ± SD. *p* < 0.01 versus TNFα alone.

Stimulation of MH7A cells with TNFα for 10 min and 1 h significantly enhanced phosphorylation of p38 MAPK and ATF-2 compared to untreated negative control cells (Fig. 6a and b). Enhanced phosphorylation of both proteins by TNFα was inhibited by co-application of HENECA (although p-p38 remained above the basal level). These findings suggest that activation of A_2A_ AdoR/Gs/AC/cAMP signaling by HENECA may reduce TNFα-mediated MMP-3 release by partially suppressing p38 MAPK and ATF-2 activation.

## Discussion

In clinical practice, serum MMP-3 levels often remain positive despite successful MTX treatment of RA, whereas serum C-reactive protein (CRP) levels fall within the reference range when MTX treatment is successful. On the basis of our results we can hypothesize that MMP-3 remains positive following successful MTX treatment due to incomplete (although substantial) suppression of TNFα-induced production by synovial cells via A_2A_ AdoR signaling.

We first confirmed that MH7A rheumatoid synoviocytes expressed all four AdoR mRNAs expressed by primary RA FLSs^11^ and that A_2A_ AdoR mRNA and cell-surface protein expression were markedly enhanced by TNFα, also in accordance with a previous report on primary RA FLSs^29^. Primary RA FLSs, however, may act differently in vitro due to differences in patient treatment history^30^, so the RA FLS line MH7A was chosen for this study.

This study focused on A_2A_ AdoR for several reasons. First, it has been reported that MTX treatment with A_2A_R-knockout mice did not show any anti-inflammatory effect^31^. Next, the expression of A_2A_ AdoR mRNA was dramatically enhanced by 1000 pg/ml TNFα, while expression levels of other AdoR mRNAs were not altered (Fig. 1a). Furthermore, A_2A_ AdoR is coupled to AC via Gs and thus activation results in intracellular cAMP accumulation and ensuing PKA signaling (Fig. 3), which is reported to suppress MMP-3 production in human chondrocytes^25^. Alternatively, A_1_ and A_3_ AdoRs are coupled to AC via Gi, leading to reduced cAMP. The selective A_2A_ AdoR agonist HENECA partially suppressed TNFα-stimulated MMP-3 production (Fig. 4), a response mimicked by the cell-permeable cAMP analog dbcAMP (Fig. 5). This apparent inhibitory effect was not due to HENECA, DMSO, or dbcAMP cytotoxicity, as none of these agents reduced viable cell number (data not shown). Moreover, the inhibitory effect of HENECA was significantly blocked by the selective A_2A_ AdoR antagonist ZM241385 (Fig. 5a) or adenylate cyclase inhibitor SQ22536 (Fig. 5b), confirming that A_2A_ AdoR is the primary mediator of reduced TNFα-stimulated MMP-3 production.

We then provide evidence that this suppressive effect of A_2A_ AdoR activation stems from partial inhibition of p38 MAPK/ATF-1 signaling. Inflammatory cytokines including TNFα are known to induce rapid activation of the MAPK signaling pathway, and several studies have reported that inhibition of MAPK phosphorylation suppresses MMP-3 production^32,33^. Further, p38 MAPK signaling is reportedly also involved in MMP-3 production by MH7A cells^34^. Expression levels of the AP-1 family transcription factor ATF-2 were higher in FLSs from RA patients than non-patients^35^ and phosphorylation of p38 and ATF-2 drives the production of MMP-3^36,37^. HENECA partially inhibited TNFα-induced p38 and ATF-2 phosphorylation (Fig. 6), in accordance with HENECA-mediated partial inhibition of MMP-3 production by TNFα.

In RA, blood cell components such as macrophages and lymphocytes infiltrate joints and induce persistent synovitis through secretion of inflammatory cytokines, which leads to the production of MMP-3^23,38^. In contrast, Gs-coupled A_2A_ AdoRs increase intracellular cAMP and suppress inflammation^39,40^. However, activation of A_2A_ AdoR signaling alone using HENECA did not reduce TNFα-induced MMP-3 production to basal levels, which may explain why MTX usually decreases but does not eliminate serum MMP-3^41^. Shiozawa et al. reported that 87.0% of RA patients treated with MTX for 3 years still exhibited blood MMP-3 levels above 103.7 mg/ml as well as ongoing joint destruction (defined as a change in van der Heijdi modified total Sharp score ≥ 3.0)^26^. They also revealed that 94.3% of patients treated with MTX and still exhibiting rapid radiographic progression (defined as a change in van der Heijdi modified total Sharp score ≥ 5.0) had blood MMP-3 concentrations above 103.7 mg/ml^26^. Similarly, Ma et al. reported that patients with continuously elevated serum MMP-3 levels for 3–6 months had showed radiographic progression even when the therapeutic target, including CRP levels, was achieved^42^. Their prospective cohort study also showed that the serum MMP-3 level was significantly higher in progressive patients than in nonprogressive patients for an entire year and that elevated serum MMP-3 levels at baseline and the first, third, and sixth months were significant predictors of 1-year radiographic progression with cutoff points of 159 ng/ml, 264 ng/ml, 178 ng/ml, and 161 ng/ml, respectively^42^. Furthermore, matrix metalloproteinase inhibitors prevented the progression of joint destruction in rats with collagen-induced arthritis^43,44^. These findings suggest that residual MMP-3 production is a crucial contributor to further joint destruction in MTX-treated RA patients.

The results presented here show for the first time the important contribution of FSL A_2A_ AdoRs signaling to the therapeutic mechanisms of MTX. These findings may also explain, at least in part, why MTX treatment alone does not normalize blood MMP-3 in most RA patients, and suggest that additional anti-TNFα treatments may be necessary to achieve long-term remission.

In conclusion, adenosine signaling via A_2A_ AdoRs, AC, and cAMP reduces (although does not completely block) TNFα-induced MMP-3 production, by interfering with p38 MAPK/ATF-2 activity. Activation of A_2A_ AdoR pathway and suppression of MMP-3 release may explain the antirheumatic effects of methotrexate.

## Methods

### Chemicals

The selective A_2A_ AdoR agonist 2-hexynyladenosine-5′-*N*-ethylcarboxamide (HENECA) was purchased from Abcam (Cambridge, UK) and TNFα from R&D Systems (Minneapolis, MN). The selective A_2A_ AdoR antagonist 4-(2-(7-amino-2-(furan-2-yl)-[1,2,4]triazolo[1,5-a][1,3,5] triazin-5-ylamino)ethyl)phenol (ZM241385) and the membrane-permeable N6,2′-O-dibutyryl cAMP (dbcAMP) were purchased from Sigma Aldrich (St. Louis, MO). The adenylate cyclase inhibitor 9-(tetrahydrofuran-2-yl)-9 h-purin-6-amine (SQ22536) was purchased from TCI (Tokyo, Japan). TNFα, dbcAMP, and SQ22536 were dissolved in de-ionized water while HENECA and ZM 241385 were dissolved in dimethyl sulfoxide (DMSO) for cellular administration.

### Cell culture

MH7A cells were obtained from Riken Cell Bank (Saitama, Japan) and cultured in RPMI 1640 medium (Thermo Fisher Scientific, Tokyo, Japan) supplemented with 10% heat-inactivated fetal bovine serum (Thermo Fisher Scientific) and 4 × Antibiotic–Antimycotic liquid (Thermo Fisher Scientific) at 37 °C under an atmosphere of 5% CO_2_.

### Cyclic AMP assay

MH7A cells were seeded at 1.0 × 10^6^/well on flat-bottomed 24-well microplates. After 90 min, cells were treated with HENECA (0–250 nM) for 0–60 min. Intracellular cAMP was measured using a cAMP EIA System (GE Healthcare, Buckinghamshire, UK) according to the manufacturer’s protocol.

### Measurement of adenosine receptor and MMP-3 mRNA expression

MH7A cells were seeded at 4 × 10^5^ /well on flat-bottomed 6-well microplates and cultured for 24 h. Cells were then treated for an additional 24 h with TNFα (0–1000 pg/ml) or HENECA (0–100 nM). Total RNA was extracted from MH7A cells using an RNeasy Mini kit and QIAshredder (QIAGEN, Tokyo, Japan) according to the manufacturer’s instructions. Aliquots of RNA were reverse transcribed using a Transcriptor Universal cDNA Master (Roche Diagnostics K.K., Tokyo, Japan), and PCR reactions performed using TaqMan Universal PCR Master Mix (Thermo Fisher Scientific) according to the manufacturer’s protocol on a LightCycler 480 instrument with LightCycler 480 Gene Scanning software version 1.5 (https://lifescience.roche.com/global_en/products/lightcycler14301-480-software-version-15.html) (NIPPON Genetics, Tokyo, Japan). The primers used for real-time PCR were as follows: A1 AdoR (Hs00181231_m1), A_2A_ AdoR (Hs00169123_m1), A_2B_ AdoR (Hs00386497_m1), A3 AdoR (Hs01560269_m1), MMP-3 (Hs00968305_m1), and GAPDH (Hs02758991_g1) (Thermo Fisher Scientific). The amplification protocol consisted of 10 min at 95 °C followed by 55 cycles of 15 s at 95 °C, 1 min at 60 °C, and 1 s at 72 °C. Expression of MMP-3 mRNA was normalized to GAPDH mRNA expression.

### Western blot analysis

Primary polyclonal antibodies against human adenosine receptor A_2A_ were purchased from Abcam, while antibodies against human phospho-p38 mitogen-activated protein kinase (p-p38 MAPK), human phospho-activating transcription factor 2 (p-ATF-2), and ß-actin was purchased from Cell Signaling Technology (Tokyo, Japan). MH7A cells were seeded on 6-cm dishes at 1 × 10^6^/dish for 24 h, then treated for an additional 24 h with TNFα (0–1000 pg/ml) (R&D Systems). Membrane- and cytoplasmic-protein fractions were extracted using the Mem-PER Plus Membrane Protein Extraction Kit (Thermo Fisher Scientific), while total cellular protein was extracted using the M-PER Mammalian Protein Extraction Reagent (Thermo Fisher Scientific). The protein concentrations in each sample were measured using a BCA Protein Assay Reagent Kit (Thermo Fisher Scientific). Proteins were separated at 10–35 μg per gel lane by SDS-PAGE according to standard protocols and transferred onto PVDF-nylon membranes (Merck Millipore, Tokyo, Japan). Membranes were blocked with 5% non-fat milk in tris-buffered saline containing 0.1% Tween-20 (TBST) for 1 h at room temperature. After a brief wash, membranes were incubated overnight at 4 °C with rabbit polyclonal antibodies against adenosine receptor A_2A_ (1:1000), p-p38 MAPK (1:1000), p-ATF-2 (1:500), and ß-actin (1:1000). Blotted membranes were then washed 3 times with TBST and incubated in horseradish peroxidase (HRP)-conjugated donkey-α-rabbit IgG (1:2500, ECL Western Blotting Detection System, GE Healthcare) for 60 min at room temperature. Protein bands were captured, digitized, and quantified using ImageQuant LAS4000 mini (GE Healthcare).

### Measurement of total MMP-3 secretion

MH7A cells were seeded on flat-bottomed 24-well microplates at 8 × 10^4^/well. After 24 h, cells were treated for an additional 24 h with TNFα (25 pg/ml) alone or in combination with dbcAMP (0–100 μM) or HENECA (0–100 nM) as indicated. Supernatant MMP-3 concentrations were measured using Panacurea MMP-3 (Sekisui Medical, Tokyo, Japan).

### Statistical analysis

All experiments were repeated at least three times, and representative results are shown. Data are presented as mean ± standard deviation (SD). Treatment group means were compared using two-tailed unpaired t-tests. A *P* < 0.05 (two-tailed) was considered significant for all tests.

## Supplementary Information

Supplementary Figure legends.

Supplementary Figure 1.

Supplementary Figure 2.

Supplementary Figure 3.

Supplementary Figure 4a.

Supplementary Figure 4b.

Supplementary Figure 5.

Supplementary Figure 6a.

Supplementary Figure 6b.

## Data Availability

The data that support the findings of this study are available from the corresponding author upon reasonable request.
