# Supplementary material for: Adenosine inhibits TNFα-induced MMP-3 production in MH7A rheumatoid arthritis synoviocytes via A2A receptor signaling
Source: Sci Rep. 2022 Apr 11;12:6033. doi: 10.1038/s41598-022-10012-6 (PMC9001689; doi:10.1038/s41598-022-10012-6)
Supplement: Supplementary file 1 — Supplementary Figure legends. [file 41598_2022_10012_MOESM1_ESM.docx]

**Supplementary Figure legends**

**Supplementary Figure S1:**

Expression of adenosine A_2A_ receptors in MH7A cells and enhancement by TNFα. MH7A synoviocytes were incubated for 24 h with the indicated concentrations of TNFα. A_2A_ Adenosine receptor (AdoR) protein in the cytosol and the membrane fraction was then determined by Western blotting, respectively. A_2A_ AdoR membrane protein expression was enhanced by TNFα in dose-dependent manner.

**Supplementary Figure S2:**

Detection of ß-actin in MH7A cells.

ß-actin was used as an internal control for Supplementary Figure S1. TNFα did not affect the expression of ß-actin.

**Supplementary Figure S3:**

Stimulation of A_2A_ AdoRs reverses the TNFα-mediated activation of p38 MAPK signaling in MH7A cells

Activation of A_2A_ receptor signaling suppressed TNFα-induced activation of p38 MAPK. Cells were incubated for 10 min in TNFα (1 ng/ml) with or without HENECA (1 μM). Expression levels of p-p38 was estimated by western blotting and densitometry. HENECA suppressed TNFα-induced phosphorylation (activation) of p38 MAPK.

**Supplementary Figure S4:**

Detection of ß-actin in MH7A cells.

ß-actin was used as an internal control for Supplementary Figure S3. Exposure time is 3 minutes (a) and 6 minutes (b). TNFα did not affect the expression of ß-actin.

**Supplementary Figure S5:**

Stimulation of A_2A_ AdoRs reverses the TNFα-mediated activation of AP-1 in MH7A cells.

Activation of A_2A_ receptor signaling suppressed TNFα-induced activation of ATF-2. Cells were incubated for 1 h in TNFα (1 ng/ml) with or without HENECA (1 μM). Expression levels of p-ATF-2 was estimated by western blotting and densitometry. HENECA suppressed TNFα-induced phosphorylation (activation) of ATF-2.

**Supplementary Figure S6:**

Detection of ß-actin in MH7A cells.

ß-actin was used as an internal control for Supplementary Figure S5. Exposure time is 3 minutes (a) and 6 minutes (b). TNFα did not affect the expression of ß-actin.
