# Supplementary figures and images for: Adenosine inhibits TNFα-induced MMP-3 production in MH7A rheumatoid arthritis synoviocytes via A2A receptor signaling
Source: Sci Rep. 2022 Apr 11;12:6033. doi: 10.1038/s41598-022-10012-6 (PMC9001689; doi:10.1038/s41598-022-10012-6)

**Cytosol**

**Membrane**

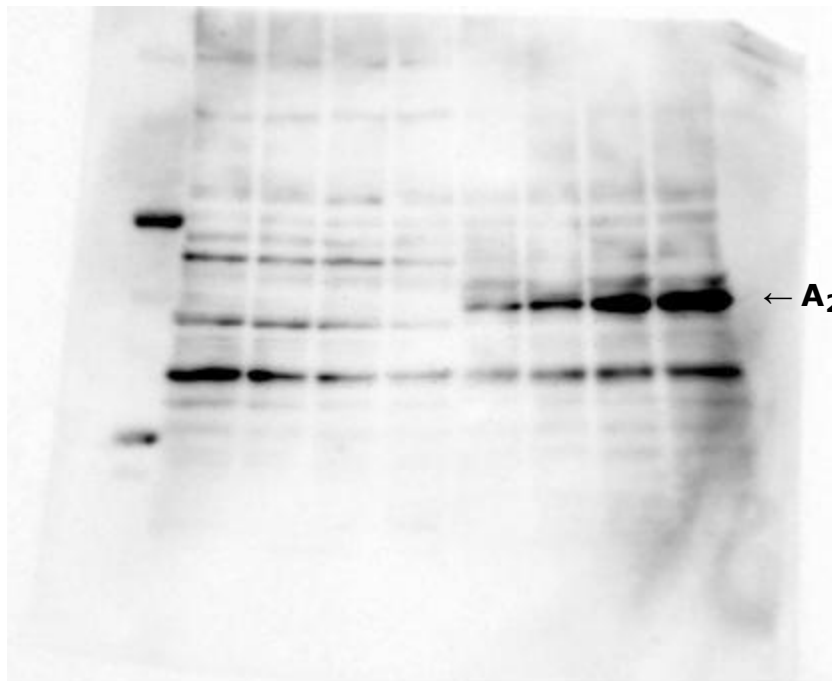

**0 50 100 1000**  
**TNFα (pg/ml)**

Supplement: Supplementary file 2 — Supplementary Figure 1. [file 41598_2022_10012_MOESM2_ESM.pdf]

**Cytosol**

**Membrane**

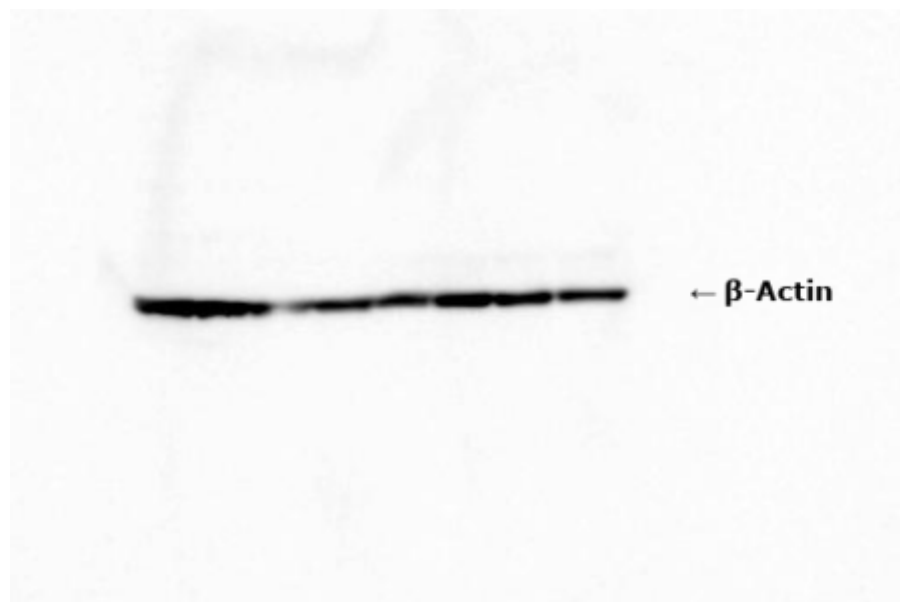

**0 50 100 1000**  
**TNFα (pg/ml)**

Supplement: Supplementary file 3 — Supplementary Figure 2. [file 41598_2022_10012_MOESM3_ESM.pdf]

**TNF $\alpha$**   
**+**  
**Control TNF $\alpha$  HENECA**

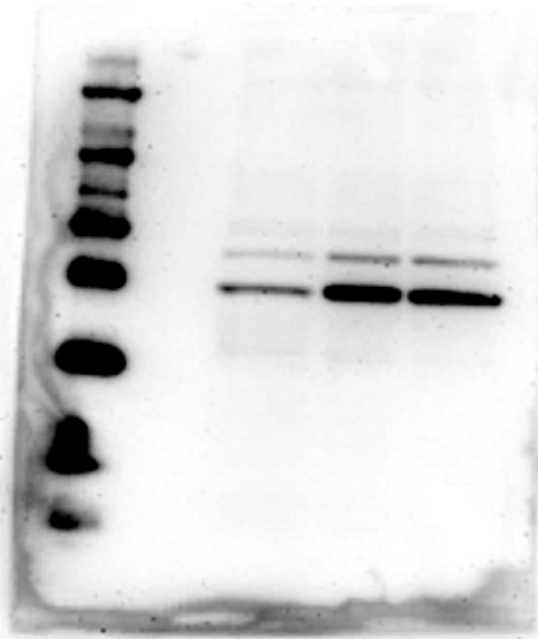

← **p-p38**

Supplement: Supplementary file 4 — Supplementary Figure 3. [file 41598_2022_10012_MOESM4_ESM.pdf]

**TNF $\alpha$**   
**+**  
**Control TNF $\alpha$  HENECA**

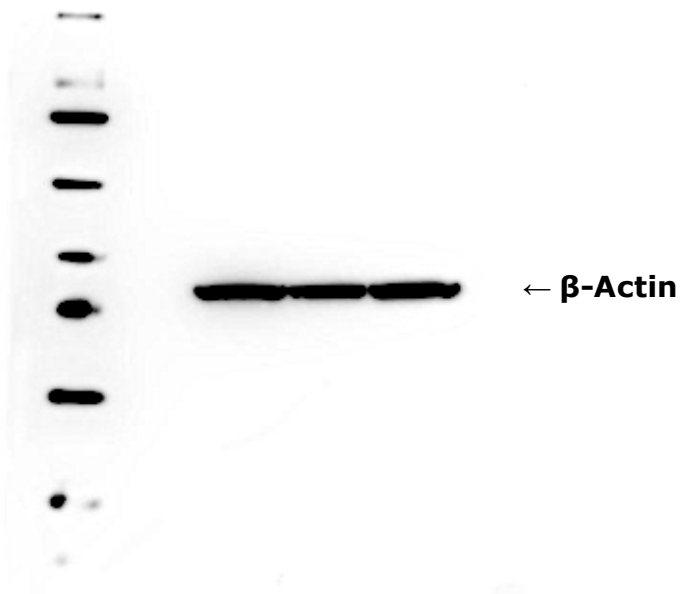

Supplement: Supplementary file 5 — Supplementary Figure 4a. [file 41598_2022_10012_MOESM5_ESM.pdf]

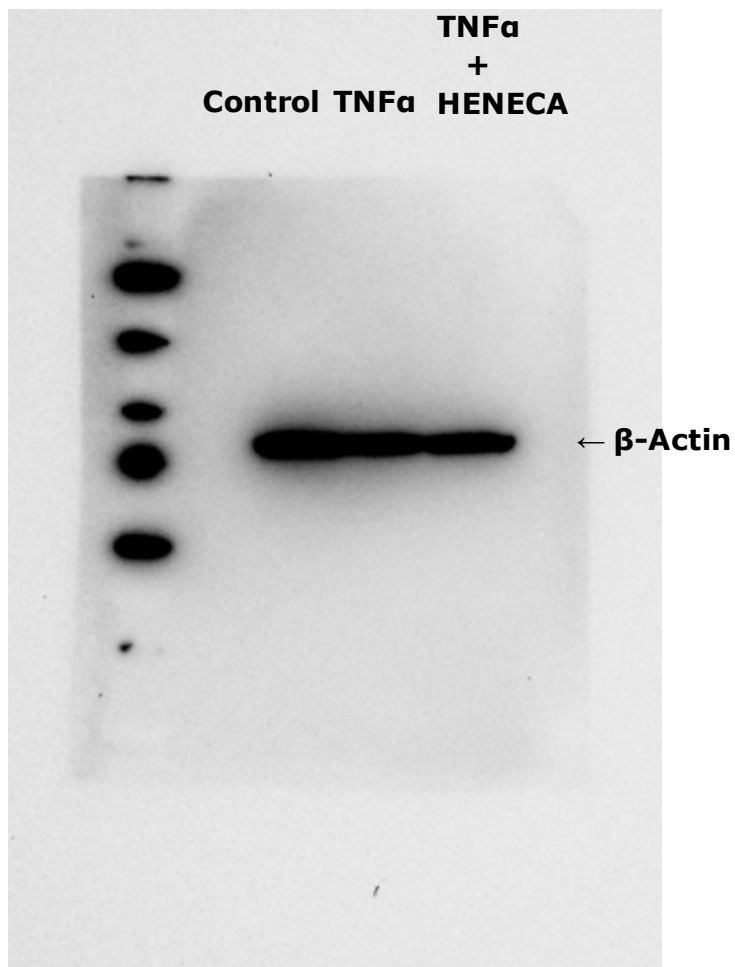

Supplement: Supplementary file 6 — Supplementary Figure 4b. [file 41598_2022_10012_MOESM6_ESM.pdf]

TNF $\alpha$   
+  
Control TNF $\alpha$  HENECA

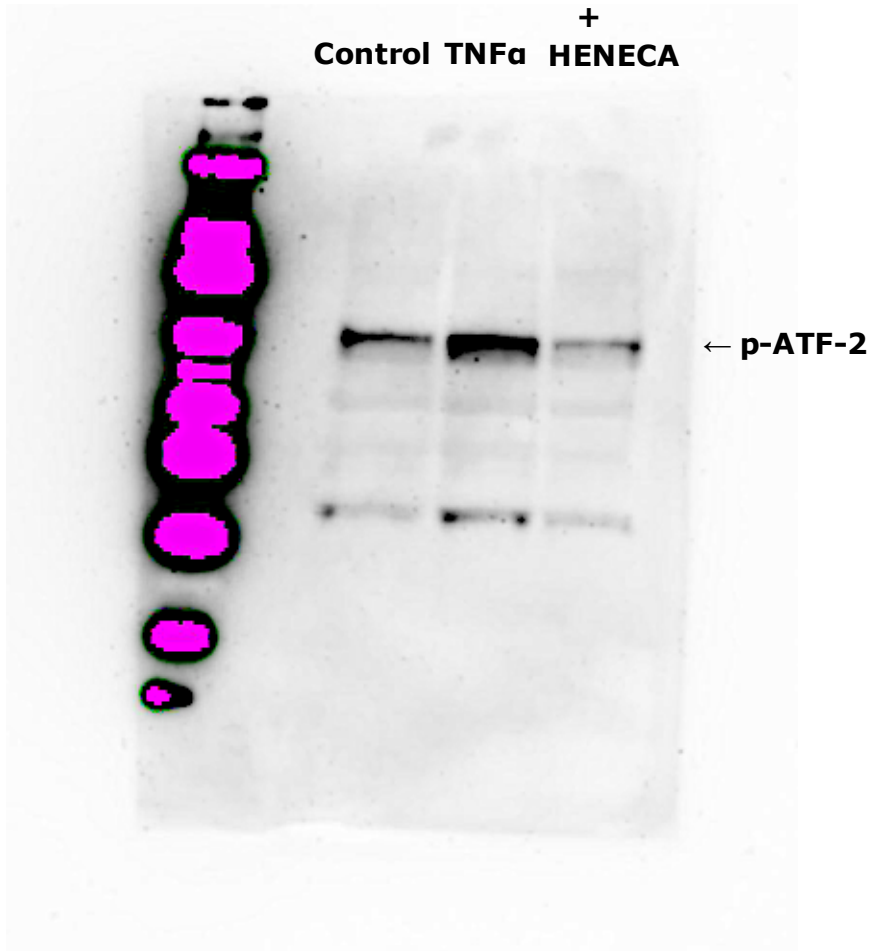

Supplement: Supplementary file 7 — Supplementary Figure 5. [file 41598_2022_10012_MOESM7_ESM.pdf]

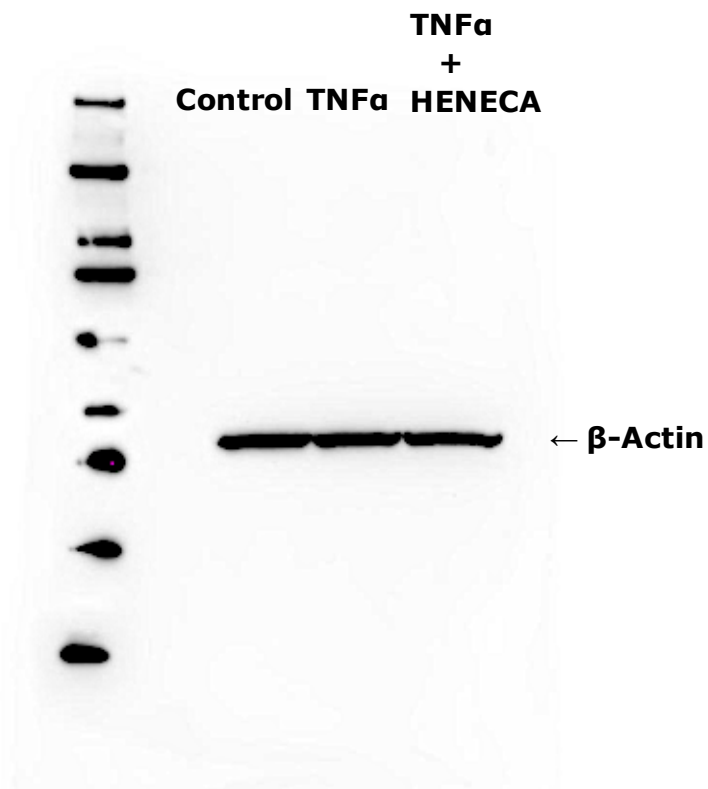

Supplement: Supplementary file 8 — Supplementary Figure 6a. [file 41598_2022_10012_MOESM8_ESM.pdf]

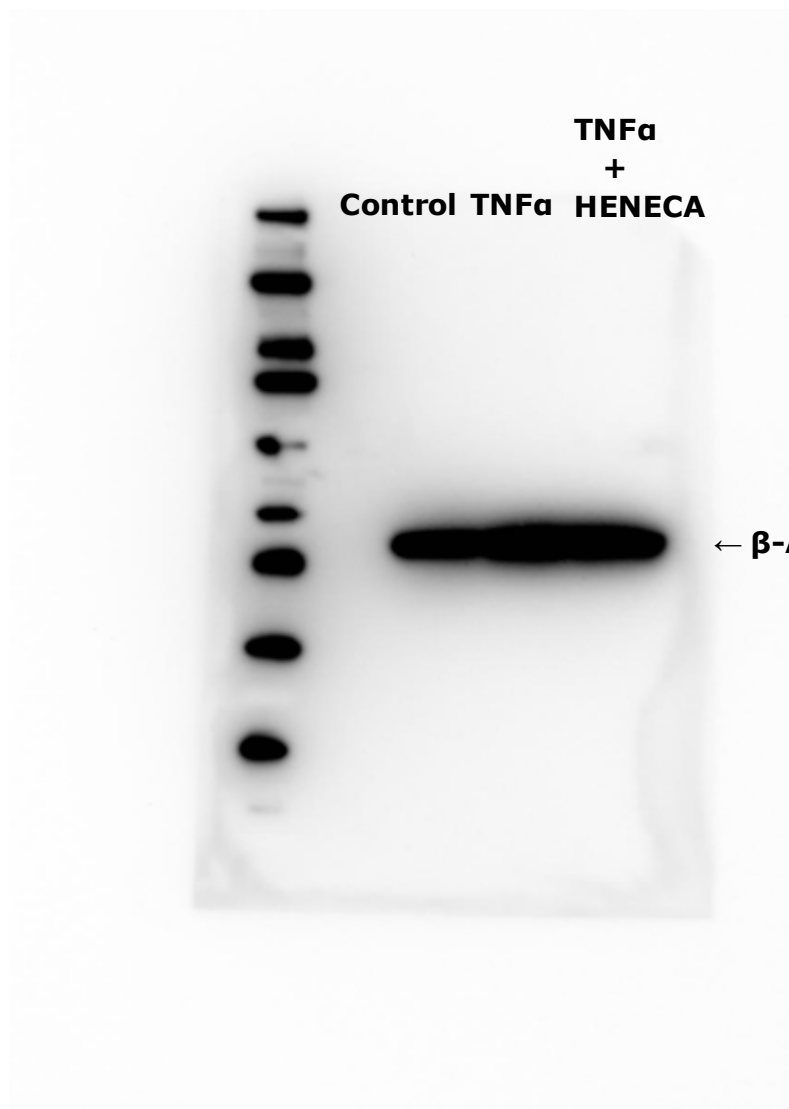

Supplement: Supplementary file 9 — Supplementary Figure 6b. [file 41598_2022_10012_MOESM9_ESM.pdf]
